# Natural strategies for creating non-equilibrium morphology with self-repairing capability towards rapid growth of excellent YBa_2_Cu_3_O_7−δ_ crystals

**DOI:** 10.1107/S2052252523000076

**Published:** 2023-01-25

**Authors:** Yanhan Zhu, Yi Yang, Xiafan Gu, Qiang Gao, Pavel Diko, Xin Yao

**Affiliations:** a Key Laboratory of Artificial Structures and Quantum Control (Ministry of Education), School of Physics and Astronomy, Shanghai Jiao Tong University, Shanghai 200240, People’s Republic of China; b Department of Materials Physics, Institute of Experimental Physics, Slovak Academy of Science, Watsonova 47, Košice 04001, Slovakia; Sun Yat-Sen University, China

**Keywords:** high-*T*_c_ superconductors, self-repair, crystal growth, non-equilibrium morphology, microstructure formation mechanisms

## Abstract

Self-repair, as a natural phenomenon, has been vastly observed and investigated in a variety of fields. With such an ability, living species self-heal their wounds to restore physiological functions while non-biological materials return to their original states, for example, thin surface layer growth occurs in the regeneration of incomplete KH_2_PO_4_ crystals. Here, two seeding strategies are developed for creating incomplete crystallographic shapes (*i.e.* right-angled concave corners) of YBa_2_Cu_3_O_7−δ_ (YBCO) superconducting crystals with self-repairing capability in top-seeded melt growth. One involves *in situ* self-assembly seeding, by which the ability to self-repair promotes YBCO growth; the other is vertically connected seeding, by which self-repair triggers YBCO nucleation. Consequently, rapid surface crystallization originated at concave corners and swiftly generated initial growth morphology approaching equilibrium. Furthermore, these rapid-growth regions including the concave crystal or seed innately functioned as sizable effective seeding regions, enabling the enlargement of the *c*-oriented growth sector and the enhancement of properties for YBCO crystals. This work demonstrates experimentally that biaxial-in-plane-aligned crystals and precisely perpendicular-arranged seeds are important self-repairing activators for the rapid growth of YBCO crystals. This nature-inspired self-repairing work offers insights into the design of seeding architecture with non-equilibrium morphology for inducing sizable high-performance crystals in the YBCO family and other functional materials.

## Abstract 2

The self-repairing nature of rapid YBa_2_Cu_3_O_7−δ_ growth with overgrowing features is revealed and non-equilibrium morphology is demonstrated to be a prerequisite self-repairing activator.

## 1. Introduction

**Figure 1:**
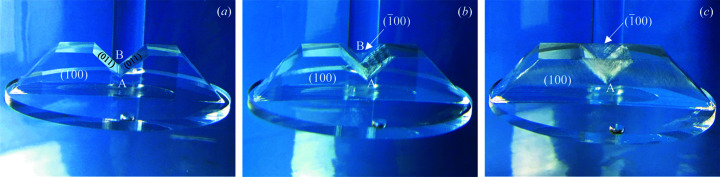
TSLG in the regeneration of a KDP crystal with a concave part. (*a*) Initial incomplete morphology. (*b*) Owing to self-repair, TSLG originates from concave corners A and B. (*c*) Restored (100) and (100) faces with thin surface layers. Figure reproduced from Wang *et al.* (2019 ▸) with permission from Wiley.

Crystal growth mechanisms have been intensively studied for more than a century, which greatly helps scientists to understand and even design the growth process. Non-equilibrium (Bunn & Emmett, 1949 ▸; Ben-Jacob & Garik, 1990 ▸; Galenko & Ankudinov, 2019 ▸; Amoorezaei *et al.*, 2012 ▸; Zaitseva & Carman, 2001 ▸), as a special crystal state that can transiently appear but does not last for long in growth, is of great importance in initiating a swift evolution in morphology, potentially leading to rapid crystal growth (Bunn & Emmett, 1949 ▸; Zaitseva & Carman, 2001 ▸). In the last few decades, non-equilibrium morphology related to the KH_2_PO_4_ (KDP) crystal was widely investigated (Zaitseva & Carman, 2001 ▸; Wang *et al.*, 2019*a* ▸,*b* ▸). One well known study often cited in KDP research is the peculiar phenomenon of thin surface layer growth (TSLG), which was amazingly observed on incomplete crystals [typically involving concave parts, see Fig. 1 ▸(*a*)]. As demonstrated in Figs. 1 ▸(*b*) and 1 ▸(*c*), once the crystal with concave (100) faces was introduced into solution, thin surface layers [dozens of micrometres thick (Wang *et al.*, 2019*a* ▸)] rapidly advanced upward and restored the (100) faces from points A and B, dominating the layer-by-layer growth on two (011) planes of the valley. In brief, TSLG is characterized by rapid film-forming and the absence of pre-existing layers (Zaitseva & Carman, 2001 ▸; Wang *et al.*, 2019*a* ▸,*b* ▸). Regarded as ‘the biggest mystery’ (Zaitseva & Carman, 2001 ▸), the mechanism of this unique growth phenomenon has not been comprehensively clarified to date. Fundamentally, it is a self-repairing process (Wang *et al.*, 2019*b* ▸), in which a crystal with incomplete parts is naturally and swiftly completed to its equilibrium shape in a supersaturated solution. In fact, self-repair is a more universal phenomenon than TSLG and has been widely found in nature in extraordinary tendencies towards restoring physiological functions for living species or returning to equilibrium states for non-biological materials (Ceratti *et al.*, 2018 ▸; Singh *et al.*, 2018 ▸).

More interestingly, TSLG was involved in the process of joining ‘two equally oriented crystals with close shape and size’ into a single crystal (Zaitseva & Carman, 2001 ▸). In that case, two grown KDP crystals with the same orientation were glued together in an alignment whereby two (101) faces were parallel to each other. After introduction into a supersaturated solution, two crystals advanced independently into a joined crystal which had a (101) common face with a concave part. Eventually, a thin surface layer rapidly formed from the concave angle to complete the face with its correct crystallographic shape.

**Figure 2:**
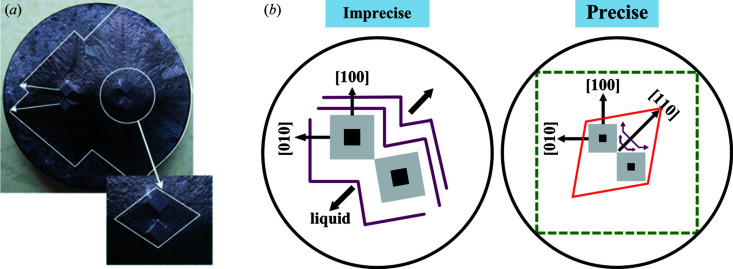
(*a*) Photographs of the top view of a GdBCO crystal induced by two sets of (110)/(110) arranged double-seeds. From the left set, two crystals with concave joints independently propagated at a normal rate, while the right-set-induced crystals combined into a large single crystal with a rapid-growth zone, as amplified in the inset (white diamond frame). (*b*) Schematic illustrations of growth modes corresponding to imprecisely and precisely (110)/(110) arranged double-seeding.

Similar joined-crystal-activated TSLG was apparent in the fabrication of REBa_2_Cu_3_O_7−δ_ (RE123 or REBCO, where RE = rare earth elements such as Y, Gd and Nd) superconducting crystals (Cheng *et al.*, 2013 ▸), which have considerable potential magnetic applications (Tomita & Murakami, 2003 ▸; Hari Babu *et al.*, 2005 ▸; Shiohara & Endo, 1997 ▸; Zhu *et al.*, 2020*a* ▸; Murakami, 2000 ▸; Werfel *et al.*, 2012 ▸). Because of the low growth rate, the rapid *ab* plane coverage of REBCO crystals in top-seeded melt-growth (TSMG) has been essential for pursuing large sizes and high performance (Cheng *et al.*, 2013 ▸). As one of the effective approaches, double-seeds are widely used to save time (Cheng *et al.*, 2013 ▸; Li *et al.*, 2010 ▸; Shi *et al.*, 2013 ▸; Kim *et al.*, 2001 ▸). Arranging two sets of (110)/(110) aligned double-seeds on a GdBCO sample with more or less uncertainty, Cheng *et al.* (2013 ▸) attained one precisely aligned set accidentally, which induced a larger crystal as shown in Fig. 2 ▸(*a*) on the right-hand side. A potential seeding/growth mode was suggested. First, the precisely arranged (110)/(110) double-seeds produce (110)/(110) double-crystals with the same precision shown on the right-hand side of Fig. 2 ▸(*b*). Then, spreading their growth fronts, these two crystals encounter on a small edge-to-edge strip and join into one crystal. Note that the newly joined crystal features an incomplete shape with innately right-angled concave corners, from which the rapid surface layer growth tends to arise. Next, the YBCO phase speedily crystallizes to form a characteristic diamond-shaped convex morphology. For this rapid-growing step, we believe that this is the same growth mechanism as the TSLG phenomenon in KDP crystals. After that, the second rapid-morphology-change from the diamond shape to a square equilibrium shape takes place, creating a complete homogenous-combined crystal. In contrast, as a right-angled concave corner could not be generated, poorly arranged (110)/(110) double-seeds, as shown in Figs. 2 ▸(*a*) and 2 ▸(*b*) on the left-hand side, induce normal growth for the two crystals involved with concaves preserved at joints of growth fronts from the beginning to the end.

In brief, taking advantage of the TSLG phenomenon related to crystal self-repair, precisely (110)/(110) aligned double-seeds could be exploited to create incomplete crystallographic morphology for originating rapid growth in the fabrication of sizable REBCO crystals. However, by employing the prior art hand-operated double-seeding techniques (Cheng *et al.*, 2013 ▸; Li *et al.*, 2010 ▸; Shi *et al.*, 2013 ▸; Kim *et al.*, 2001 ▸), seeds were fabricated *ex situ*, arranged and then heated, and hence were unavoidably subject to misalignment. Unlike the demonstrative work of precisely (110)/(110) arranged seeding in GdBCO crystals as introduced above, what we pursue here is the realization of precise seeding alignment for creating right-angled concaves and the complete growth of YBCO crystals. For this aim, two approaches were developed by seeding assembly design for attaining non-equilibrium morphology, which could effectively and reliably induce rapid *ab* plane growth of REBCO crystals.

## 2. Experimental methods

YBCO single-domain crystals were produced in air by a so-called cold-seeding TSMG method (Zhu *et al.*, 2020*a* ▸) utilizing both conventional precursor powder (CPP) and modified precursor powder [MPP (Liu *et al.*, 2017 ▸) for potentially higher properties]. First, three starting materials YBa_2_Cu_3_O_7−δ_ (Y123), Y_2_BaCuO_5_ (Y211) and Ba_2_Cu_3_O_*x*_ (Y023) were obtained through solid-state reactions by mixing Y_2_O_3_ (Y200), BaCO_3_ and CuO powders in stoichiometric ratios. The fully mixed raw powder was calcined at 900°C for 48 h and then the process was repeated three times to ensure its high purity. Second, CPP was obtained by mixing Y123 and Y211 in the molar ratio Y123:Y211 = 1:0.3 and MPP was obtained by mixing Y200 and Y023 in the molar ratio Y200:Y023 = 0.8:1.1. Additionally, 1 wt% of CeO_2_ was added to both CPP and MPP mixtures.

For *in situ* self-assembly (ISSA) seeding, MPP was pressed into three kinds of pellets with different diameters (Φ) and heights (H): one main pellet (Φ = 30 mm, H = 14 mm), one main buffer (Φ = 10 mm, H = 1.5 mm) and two mini-buffers (Φ = 5 mm, H = 1 mm). Then we adopted a novel film-seed/main-buffer/mini-buffers construction that would be deposited on the top surface of main pellet. A commercial 2 × 2 mm *c* axis-oriented Y123-buffered NdBCO thin-film seed [shortly, a NdBCO film-seed (Chen *et al.*, 2015 ▸; Tang *et al.*, 2005 ▸)] was placed on the centre of main buffer to induce crystallization. The centre distance of mini-buffers was denoted the mini-buffer distance (*D*).

As for vertically connected seeding, CPP and MPP were pressed into Φ = 20 mm and H = 10 mm as main pellets. Strip-seeds of 9 × 2 mm and 4.5 × 2 mm were first sliced from a commercial 10 × 10 mm NdBCO film-seed and then bonded into T- and Z-arranged seeding patterns using ceramic glue (Aron Ceramic, type C). Then one vertically connected seed was placed on the centre of an MPP pellet to induce crystallization. For comparative study, a same-sized CPP bulk was induced by a 9 × 9 mm film-seed as a reference sample, labelled sample C9.

Also, a Y123 pellet with a Yb_2_O_3_ support (Guo *et al.*, 2015 ▸) isodiametric to the main pellet and 4 mm-thick was inserted at the bottom of each precursor as a liquid source. Finally, the preform was heated to the maximum temperature (*T*_max_, 1065°C for 20 mm main pellets and 1075°C for 30 mm pellets) over 10 h and, after being held at *T*_max_ for 1 h, was rapidly cooled to the starting temperature (*T*_start_, 1006°C). The seed-induced epitaxial growth proceeded via cooling to the end temperature (*T*_end_) at a rate of first 0.3 K h^−1^ and then 0.5 K h^−1^ within 70 h before the fully grown crystal was furnace-cooled to ambient temperature.

As-grown samples were polished for microstructural study using a scanning electron microscope (SEM, MIRA3 TESCAN) and an optical microscope (OLYMPUS BX51M). Quantitative analysis was performed using the image processing software *ImageJ*. Superconducting properties were characterized by trapped field. Before measurement, a fully grown YBCO bulk was split off its seeding construction and liquid source pellet and then annealed in flowing oxygen at a temperature of 450°C for 240 h. To test trapped field, the YBCO crystals were magnetized under a field-cooled state to 77 K with a 2 T external field parallel to the crystallographic *c* axis. The samples were stabilized over 15 min and then scanned by a Hall sensor (Lake Shore) with the sensing area being 0.7 mm above the sample.

## 3. Results and discussion

### 3.1. *In situ* self-assembled (110)/(110) twin-seeding and TSLG facilitated YBCO growth

**Figure 3:**
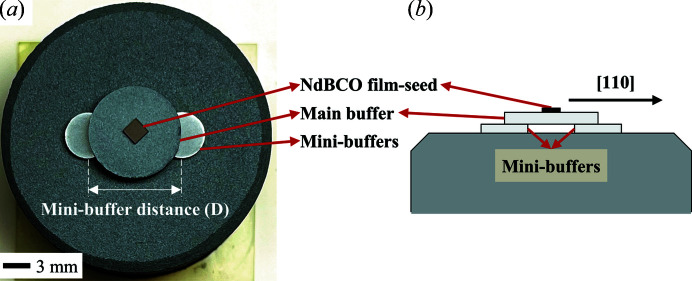
(*a*) Photograph of an initial ISSA seeding architecture with *D* = 10 mm. (*b*) Schematic showing the side view of film-seed/main-buffer/mini-buffers construction.

**Figure 4:**
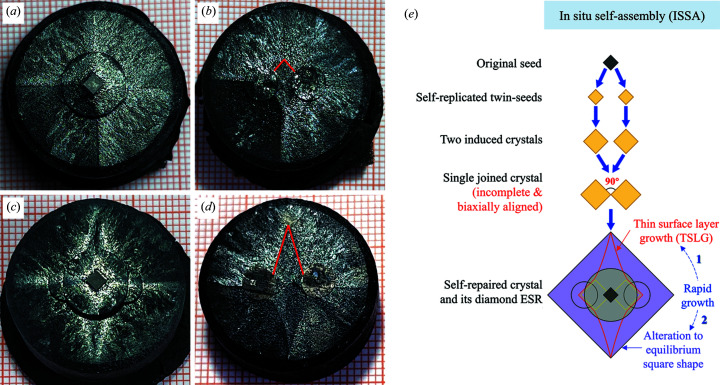
Photographs of two fully grown YBCO crystals (on millimetre square graph papers) processed by ISSA seeding: top views (*a*) and (*c*) before and (*b*) and (*d*) after splitting off their seeding constructions. Distances between mini-buffers (*D*) are 7 and 10 mm for samples in (*a*) and (*c*), respectively. (*e*) Schematic illustration of the route of ISSA seeded YBCO growth.

As for double-seeds used in TSMG processes, two types of alignment, (100)/(100) and (110)/(110), were widely investigated, verifying that (110)/(110)-arranged double-seeds could produce cleaner (110)/(110) grain boundaries (Li *et al.*, 2010 ▸; Kim *et al.*, 2001 ▸). More importantly, as stated above, it was found that the precision of (110)/(110) seeding alignment has great importance in attaining rapid growth (Cheng *et al.*, 2013 ▸). To overcome (110)/(110) misalignment problems caused by *ex situ* artificially operated real seeds, we developed a novel seeding approach for double-seeded TSMG, as shown in Fig. 3 ▸. A unique arrangement was made in this new design, in which either side of the extended diagonal line of seed (naturally [110] oriented) passed through the centre of a mini-buffer. Self-replicating the [110] crystallographic orientation from seed through main buffer, mini-buffers innately become exact (110)/(110) twin-seeds *in situ* during the thermal procedure. For this reason, we named this approach *in situ* self-assembly (ISSA) seeding. Remarkably, exploiting such exact (110)/(110) twin-seeds, for the first time, we succeeded in growing 25 mm-diameter YBCO crystals without incomplete concave parts, as shown in Figs. 4 ▸(*a*)–4 ▸(*b*) and 4 ▸(*c*)–4 ▸(*d*) for a mini-buffer distance *D* = 7 mm and 10 mm, respectively. Figs. 4 ▸(*a*) and 4 ▸(*c*) exhibit fully covered surfaces with fourfold growth facet lines from the top views of samples. More fascinatingly, after splitting off seeding constructions from as-grown samples, a distinctive quasi-diamond-shaped region is evidently observable in Figs. 4 ▸(*b*) and 4 ▸(*d*), signifying that TSLG-related rapid growth has taken place as clarified above. Instead of the demonstrative work of precise (110)/(110) alignment (Cheng *et al.*, 2013 ▸), in which the precision of the arrangement was uncontrollable and the crystal was not fully grown, this ISSA approach could reliably attain exact (110)/(110) alignment and realize complete growth of YBCO crystals.

The ISSA seeding mode is illustrated in Fig. 4 ▸(*e*). First, acting as a would-be self-template, the main buffer transmits the texture structure from the seed to mini-buffers and generates precise (110)/(110) twin-seeds *in situ*. Then, the twin-seeds induce two separate crystals with highly consistent orientations on the main pellet. Next, the induced crystals grow at a normal rate until they encounter and combine into a single but incomplete crystal with initial concave corners. After that, rapid self-repair (*i.e.* TSLG) initiates from the concaves. The YBCO phase quickly fills the gap and overgrows to form a diamond-shaped convex morphology. With this diamond growth front approaching equilibrium, it triggers the second type of rapid growth through transforming to an equilibrium-square shape. Finally, the YBCO crystal propagates in its square growth habit over time until the sample is entirely covered.

**Figure 5:**
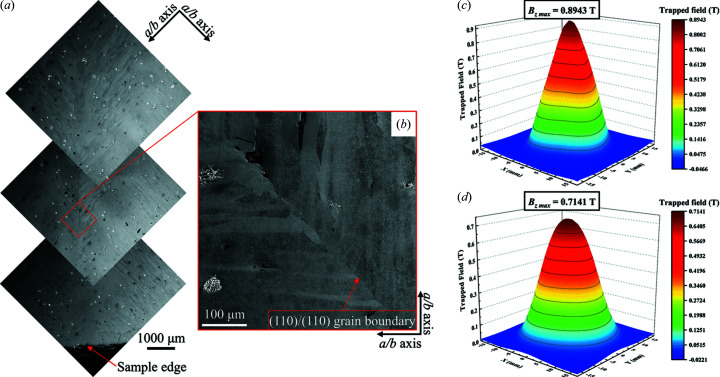
(*a*) SEM micrographs of an (110)/(110) ISSA seeded sample from the middle to the edge. (*b*) Local SEM image at a higher magnification showing the details of the (110)/(110) grain boundary. (*c*) and (*d*) Trapped field profiles (3D maps) measured at top surfaces of samples in Fig. 4 ▸(*a*) and 4 ▸(*c*).

**Figure 6:**
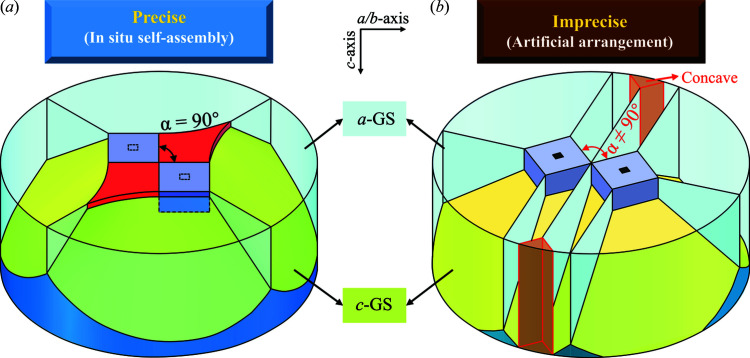
Perspective schematics showing the structures of crystals induced by (*a*) ISSA precise (110)/(110)-aligned seeding and (*b*) artificially arranged imprecise (110)/(110)-aligned seeding. Angle α indicates the in-plane angle between two (100)-sided grains (coloured purple).

Furthermore, in the TSLG mode, rapid filling of Y123 at incomplete concave corners can effectively suppress potential impurities that normally segregate in the low-speed growth process. SEM photographs in Figs. 5 ▸(*a*) and 5 ▸(*b*) exhibit a (110)/(110) grain boundary from the ISSA seeded crystal, showing an almost undetectable width and imperceptible impurities. Therefore, through self-repair, TSLG gives rise to a natural and strong connection between two growing crystals, forming a homo-combined single-domain crystal. Additionally, the diamond region transiently forms along with the initial proceeding of the *c* growth sector (*c*-GS), which, including the joined crystal, functions as a sizable effective seeding region (ESR) and results in an enlarged *c*-GS [Fig. 6 ▸(*a*)]. Both the contamination-free grain boundary and the large-sized *c*-GS are definitely beneficial to superconducting performance. As a result, corresponding to samples in Figs. 4 ▸(*a*) and 4 ▸(*c*), trapped fields of 0.8943 T and 0.7141 T with single peaks are achieved in Figs. 5 ▸(*c*) and 5 ▸(*d*), which are superior to those of the same-sized bulks in double-seeding work reported (Li *et al.*, 2010 ▸; Shi *et al.*, 2013 ▸; Kim *et al.*, 2001 ▸) and even comparable to the best results utilizing single-seeding (Tang *et al.*, 2016 ▸; Zhu *et al.*, 2020*b* ▸).

Most distinctively, the ISSA approach enables rapid *ab* plane coverage through TSLG in producing REBCO crystals. Induced by precisely aligned (110/(110) twin-seeds, the two same-aligned crystals create a biaxially oriented structure in the (001) common plane of the joined crystal, which is identical to a larger incomplete square (001) plane with two concave missing parts. Such an incomplete crystallographic morphology far from equilibrium initiates a self-repairing process of the YBCO crystal, naturally and swiftly completing its incomplete surface to an equilibrium shape. It can be concluded that a (110)/(110) biaxially in-plane-aligned structure in Fig. 4 ▸(*e*) is the essential prerequisite for the rapid self-repair in double-seeded YBCO crystals. Conversely, transmitted from imprecisely (110)/(110) arranged double-seeds, two induced-crystals are poorly aligned in the plane, due to not being in a common plane and lacking repair performance when they meet. As a consequence, the two crystals still independently spread themselves outward at a normal rate, characteristically forming six approximately parallel *a*/*a* growth sector boundaries [*a*/*a*-GSBs, the middle two are also termed (110)/(110) grain boundaries] and two concave corners at the sample edge as shown in Fig. S1 of the supporting information and Fig. 6 ▸(*b*).

Notably, in rapid surface growth morphology, unlike KDP, TSLG in YBCO not only restores the incomplete plane but also behaves in an overgrowth manner, leading to diamond fronts rather than a perfect square. This manner can be mainly attributed to the YBCO growth habit, the differences in edge lengths of two concave sides and the growth environment. Another feature of the TSLG-related morphology is that the diamond region has a downward curvature, which can be explained by the solidification shrinkage from melt to solid under the surface layer.

In brief, the simple but effective ISSA seeding architecture not only generates exact (110)/(110) twin-seed-alignment for the full growth of REBCO crystals, but also gives rise to rapid *ab* plane coverage by the self-repairing effect. This new approach innately creates a wealth of unique characteristics that are not presented in traditional double-seeding methods as follows:

(1) Precise (110)/(110) twin-seeds are attained by self-replicating seed orientation, thoroughly overcoming the misalignment-related problems, such as contaminated grain boundaries, incomplete growth surface, double trapped-field peaks and so on.

(2) With the same precision, two induced (110)/(110) crystals yield exact right-angled concaves at their joints by nature, creating non-equilibrium morphology for rapid self-repair.

(3) Through self-repair, rapid filling of Y123 at concaves forms an initial growth morphology with a peculiar diamond shape according to its growth habit, which then shifts to an equilibrium-square.

(4) This rapid-spreading behaviour intrinsically enables effective suppression of potential impurities between crystals, leading to a clean boundary and a homogenous-combination.

(5) The TSLG-derived diamond region, including the joined crystal, innately functions as a sizable effective seeding region, enabling the enlargement of *c*-GS and high trapped fields for YBCO crystals.

(6) The ISSA seeding approach principally and naturally has no mini-buffer distance limit on attaining a joined crystal for a mono-peak trapped field profile while artificially aligned double-seeding methods have a limited seed distance on attaining a single grain for one trapped field peak.

### 3.2. Vertically connected seeding and TSLG-originated YBCO nucleation

Apart from multiple-seeding methods, one more conventional strategy for large-scale REBCO crystals is the utilization of a large-sized seed (Li *et al.*, 2015 ▸; Wu *et al.*, 2009 ▸), by which a sizable *c*-GS could be directly induced (Guo *et al.*, 2015 ▸; Li *et al.*, 2015 ▸). However, a large seed means a large surface coverage, which inhibits the release of oxygen derived from peritectic melting (Sudhakar Reddy *et al.*, 2001 ▸). The residual bubbles largely accumulate as pores in the crystallized sample and are certainly detrimental to the superconducting properties (Li *et al.*, 2015 ▸). In this section, we introduce a newly developed vertically connected seeding approach for enlarging *c*-GS through rapid self-repair while maintaining an almost unchanged porosity.

**Figure 7:**
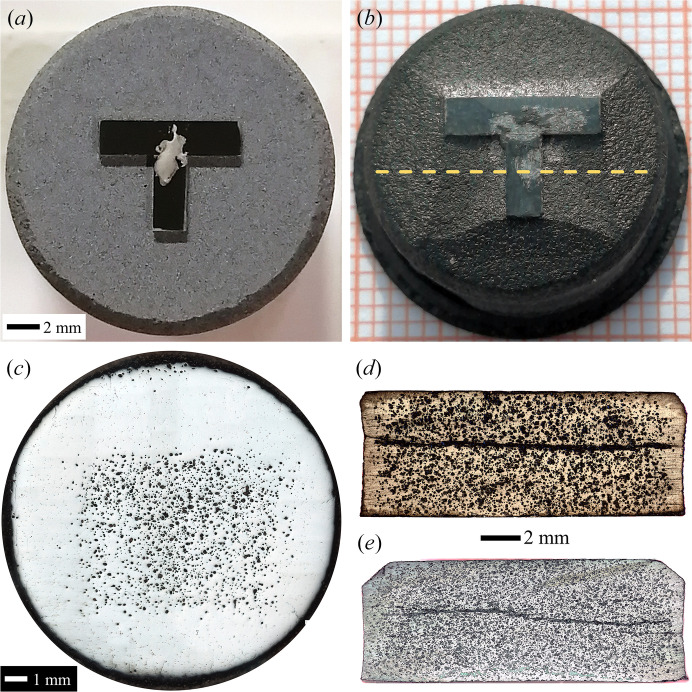
Photographs of YBCO samples by vertically connected seeding, T-type for (*a*)–(*d*) and Z-type for (*e*). Top views of (*a*) the initial precursor pellet with a T-seed and (*b*) the as-grown crystal after splitting off the T-seed. Polished samples under an optical microscope: (*c*) top view and (*d*) and (*e*) cross-sections. The scale bar is the same for (*d*) and (*e*).

As a representative of vertically connected seeding patterns (including T-, X-, L-, Z- and W-types), a T-type connected-seed (T-seed) is presented in Fig. 7 ▸(*a*), in which two strip-seeds with (100) sides are perpendicularly spliced together. In that case, the initial T-seed innately possesses right-angled concave corners, which endow it with an incomplete crystallographic morphology for activating self-repair. By applying such a T-seed in TSMG, we succeeded in fabricating 16 mm-diameter YBCO crystals (labelled sample MT). The fully covered single-domain crystal with fourfold growth facet lines in Fig. 7 ▸(*b*) indicates that the T-seeding pattern worked well. Distinctively, an initial growth morphology of a kite-like Y123 zone was identified from every corner of the T-seed, featuring convex overgrowth fronts similar to the right-angle-derived diamond region in ISSA seeding, indicating the same self-repairing manner.

**Figure 8:**
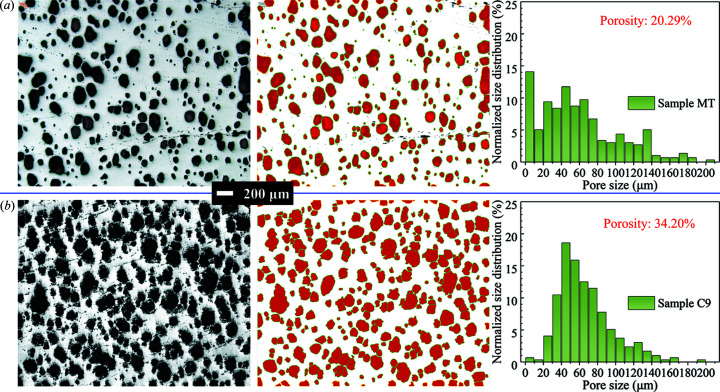
Optical micrographs (both taken at the centres of the *ac* planes), processed images by *ImageJ* and normalized pore-size distributions with porosities of YBCO crystals grown under different conditions: (*a*) MPP with a T-seed, (*b*) CPP with a 9 × 9 mm seed.

**Figure 9:**
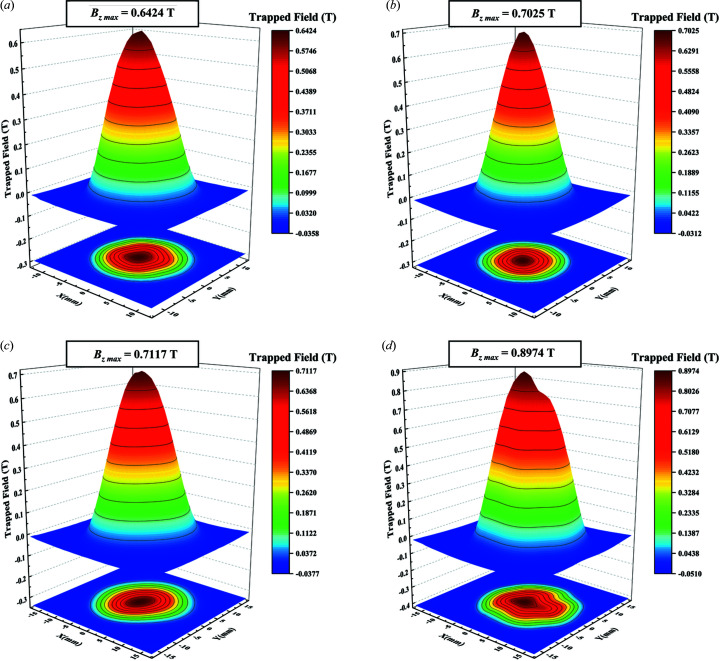
Trapped-field profiles (measured at 77 K) of samples induced by vertically connected seeds: 16 mm-diameter crystals with (*a*) T-type and (*b*) Z-type seeds, 25 mm-diameter crystals with (*c*) L-type and (*d*) T-type seeds.

Furthermore, the kite-like regions, together with the T-seed, have the same function as the diamond region in ISSA seeding, *i.e.* as a sizable ESR for generating a large *c*-GS. After polishing the top surface, a high pore density area shown in Fig. 7 ▸(*c*) is observed at the same ESR site, indicating that unreleased oxygen bubbles are trapped by a rapid-covering layer. Cutting along the yellow dashed line in Fig. 7 ▸(*b*), a large-sized *c*-GS expanding from the sizable ESR can be clearly observed from the cross-sectional image in Fig. 7 ▸(*d*) with two small *a*-GSs separated by *a*/*c*-GSBs. Since the pore structure has a great influence on superconducting properties, a comparative study between sample MT and C9 was quantitatively conducted and is exhibited in Fig. 8 ▸. Labels M and C represent MPP and CPP materials while T and 9 indicate T-connected and 9 × 9 mm seeds, respectively. Surprisingly, although the T-seed produced a sizable ESR with an approximate area to the 9 × 9 mm seed, the 20.29% porosity of MT is much lower than 34.20% of C9 and comparable to 19.77% from a small-size-seeded one (Li *et al.*, 2015 ▸). The promising microstructure of the YBCO crystal is certainly beneficial to its superconducting properties. Fig. 9 ▸ presents field trapping abilities of vertically connected seeded samples. The trapped-field profile detected from the T-patterned crystal highlights a mono-peak of 0.6424 T, which is obviously higher than the former best result of 0.636 T (Liu *et al.*, 2017 ▸) from a same-sized crystal by refining Y211 particles during TSMG. Following the identical conception, a Z-type seeding pattern was also adopted. As expected, an even more sizable *c*-GS with indiscernible *a*-GSs in Fig. 7 ▸(*e*) was induced in that crystal, resulting in a record trapped field of 0.7025 T in Fig. 9 ▸(*b*). It is promising that, by enlarging the crystal size to 25 mm in diameter, the peaks of trapped fields were further enhanced to 0.8974 T for T-pattern-related crystals and 0.7117 T for L-pattern-related crystals, as shown in Figs. 9 ▸(*c*) and 9 ▸(*d*).

**Figure 10:**
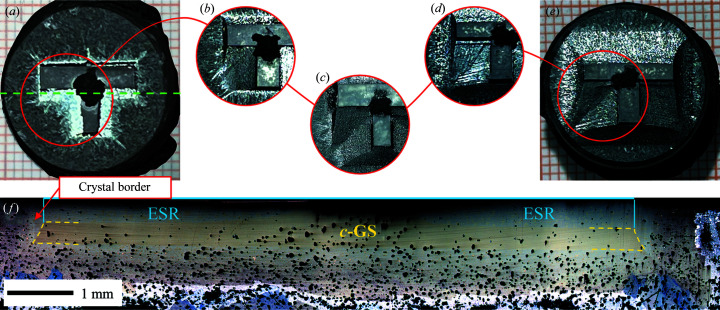
(*a*)–(*e*) Photographs showing the intermediate states of TSLG from a series of T-seeded samples. Each photograph in (*b*)–(*d*) shows one concave. (*f*) Cross-sectional image obtained by cutting the sample along the green dashed line in (*a*).

Analogous to the biaxial in-plane alignment in ISSA seeding, it is the precisely perpendicular arrangement in vertically connected seeding that ensures a common plane, serving as a self-repairing activator. The TSLG-derived initial growth morphology featuring overgrowth fronts, as one of the major YBCO self-repairing characteristics, is commonly presented in both seeding approaches. The instant-forming mode can hardly be investigated in ISSA seeding due to the difficulty in tuning starting and finishing times. Nevertheless, in vertically connected seeding, TSLG triggers YBCO nucleation, which swiftly spreads to form initial growth morphology. Therefore, it is relatively easy to catch intermediate states during morphology alteration by setting up growth time. Indeed, through the T-seeded YBCO growth, we repeatably attained such intermediate stages of self-repair in order to phenomenologically elucidate the growth mechanism. Fig. 10 ▸(*a*) shows the stage of seed-induced nucleation in which the concave edges are distinguished from other sides by highlighting larger crystallized dimensions, supporting the self-repairing ability and the rapid-growing tendency. With continuous and rapid filling, the concave becomes smaller with its shape unchanged until it vanishes [Figs. 10 ▸(*b*)–10 ▸(*d*)], presenting a convex profile approaching equilibrium. Eventually, facilitated by the second-type rapid-growth of shape alteration, an equilibrium-shaped crystal was attained in Fig. 10 ▸(*e*). Such a process is inconsistent with the previous explanation (Cheng *et al.*, 2013 ▸) that (110) planes potentially emerge and originate in the rapid-growth zone. In this T-seeded YBCO growth, only (100) faces are observed throughout the formation of kite-like regions in Figs. 10 ▸(*a*)–10 ▸(*d*), which can be attributed to the natural selection of low-energy faces. We therefore suggest that such (100) faces would advance from two concave sides and impinge, making the concaves increasingly small until both sides converge to the same point. The grain boundary is thus interpreted as a series of impingement tracks of (100) faces. Notably, due to the length difference of the two concave sides, such grain boundaries that can also be considered geometrically as the diagonal of kite-shaped regions in Figs. 7 ▸(*b*), 10 ▸(*d*) and 10 ▸(*e*) normally present a deviation from the [110] direction, which is different from [110]-oriented (110)/(110) grain boundaries in ISSA seeding.

It is clear that, based on the observation from T-seeding, such a formation mode of initial growth morphology also suits the ISSA seeded growth. Note, even if crystal spreading fronts are (100) faced in both precise and imprecise (110)/(110) seeding from this perspective, their growth habits are completely different. In the precise (110)/(110) seeding, biaxial-in-plane-aligned crystals with self-repair capability produce rapid growth. Conversely, without the self-repair property in the imprecise case, the two crystals propagate in normal growth at a much slower rate, which induces increasingly segregated impurities on the growth front, leading to incomplete growth with concave corners.

Finally, since the ESR was derived by self-repair with rapid (100)-oriented spreading, from which the *c*-GS proceeded in the [001] direction at a normal rate, these two different growth habits generate a distinguishable border. Fig. 10 ▸(*f*) presents the cross-sectional image of the T-seeded crystal, showing a noticeable boundary between two crystallized regions. The ESR with a blue frame is less than 0.5 mm thick and the *c*-GS is framed by yellow dashed lines. It is obvious that this large ESR formed *in situ* induces a sizable *c*-GS but allows more or less the release of oxygen bubbles from the sample. In short, vertically connected seeding patterns could give rise to the TSLG growth habit, creating a sizable ESR and *c*-GS with an approximately unaffected porosity and resulting in excellent superconducting properties of YBCO crystals.

### 3.3. Overview of seeding-governed rapid growth of REBCO crystals

Regarding crystal morphology, there is a general tendency for all surfaces to be low-index faces (Bunn & Emmett, 1949 ▸; Buckley, 1951 ▸). High-index faces may eliminate themselves by straightforward growth since they have higher crystallization rates than other faces (Bunn & Emmett, 1949 ▸; Buckley, 1951 ▸; Tian *et al.*, 2007 ▸). In an alternative mode, the elimination of high-index faces may occur by breaking up into steps with low-index surfaces (Bunn & Emmett, 1949 ▸). It is peculiar that, for incomplete crystallographic morphology such as concave corners, elimination arises by forming surface layers which complete the face with its correct crystallographic shape (Zaitseva & Carman, 2001 ▸; Wang *et al.*, 2019*a* ▸,*b* ▸).

**Figure 11:**
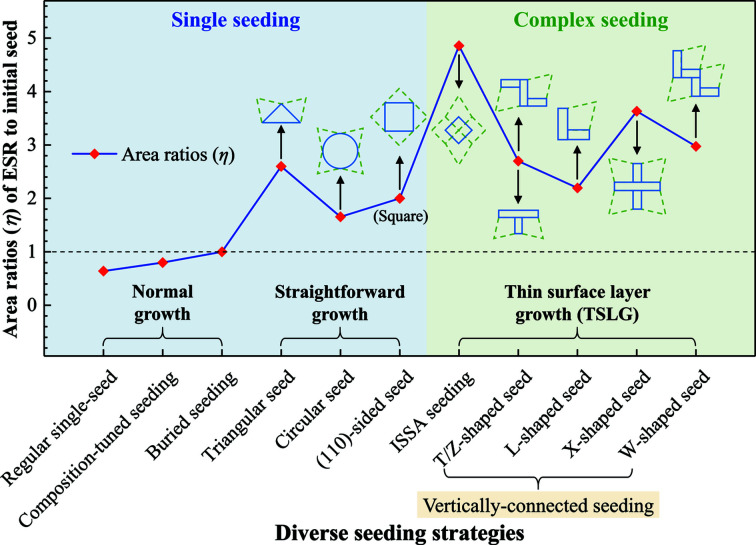
Diverse seeding strategies with their growth habits and area ratios (η) of ESR to initial seed in TSMG of REBCO superconducting crystals. A detailed calculation of η can be found in the supporting information.

As stated above, rapid growth can originate via a change from non-equilibrium to equilibrium crystal morphology. Accordingly, rapid *ab* plane coverage has been well demonstrated in the growth of REBCO crystals using non-equilibrium-shaped seeds, which would rapidly grow in a supersaturated melt, shifting to a correct crystallographic shape, and become a sizable ESR. Here, we introduce a parameter η which represents the area ratio of ESR to initial seed for signifying seeding effectiveness. An overview of diverse seeding strategies correlated with η values is presented in Fig. 11 ▸, which strongly relates to the seed orientation, seed shape, seeding assembly and seeding mode.

Normally, equilibrium-shaped square seeds are used in TSMG of REBCO crystals, in which the wetting behaviour significantly affects the seeding process as well as the η value. Due to the low wetting ability of the NdBCO film-seed with the RE–Ba–Cu–O melt, the effective contact area (ECA, equivalent to ESR) of the seed with the molten pellet is smaller than the seed area [η < 1 (Li *et al.*, 2015 ▸)]. Such wetting ability of the melt could be modified by tuning the liquid composition (Huang *et al.*, 2018 ▸). For instance, to protect the seed from the corrosive Ag-contained melt, the Ba-rich liquid was exploited to reduce the wetting ability of the seed and its ECA on the molten NdBCO/Ag pellet. Conversely, through forced wetting, ECA could also be increased by a buried seeding mode (η = 1), through which the oxygen release from the seed was suppressed and seeding thermal stability was enhanced (Xiang *et al.*, 2016 ▸).

By replacing the normal equilibrium-shaped square seed, unconventional single-seeds with irregular geometry or non-equilibrium sides have been investigated, such as triangular or circular shaped (Sudhakar Reddy *et al.*, 2005 ▸), or (110)-sided ones (Qian *et al.*, 2018 ▸; Nizhelskiy *et al.*, 2007 ▸). In Fig. 11 ▸, these three seeds commonly expose high-index (110) faces on their peripheries in part or as a whole, and are grouped in straightforward growth, through which the elimination of the (110) faces proceeds. As depicted by green dashed lines, triangular- and circular-shaped seeds enable speedy straightforward growth from high-index (110) faces, leading to trapezoid- and four-star-like initial growth morphology approaching equilibrium, respectively. Regarding the square-shaped (110)-sided seed, straightforward growth brings about a change from a high-index (110) outline to a low-index (100)- and (010)-sided contour while maintaining the same square shape (Qian *et al.*, 2018 ▸). Since these near-equilibrium shapes speedily emerge in the very beginning along with the early growth of *c*-GS, the corresponding areas function as ESRs. Note that all ESRs created by three (110)-type single seeds cover larger areas than their original seeds, *i.e.* η > 1. That is to say, irregular seeds could act as sizable seeds to induce the growth of YBCO crystals. More importantly, the second-type rapid growth tends to take place from the initial growth morphology to an ultimate equilibrium-square shape, which benefits a rapid surface coverage.

Instead of straightforward-growing irregular seeds, based on the rapid self-repairing mode, novel and complex seeding constructions, ISSA seeding and vertically connected seeding have been developed in this work. Both approaches are generally capable of creating incomplete crystallographic morphology, *i.e.* precise right-angled concave corners, for a fast coverage of the *ab* surface and then an enlargement of *c*-GS in TSMG-processed YBCO crystals. By comparing these two complex seeding approaches, there are several distinctive points. First, vertically connected seeding initiates TSLG at the YBCO nucleation stage, much earlier than ISSA seeding where TSLG would not occur until the impingement of two growing crystals which evolved from the original seed and then twin-seeds. Thus, from a time-saving point of view, the vertically connected seeding might be more efficient. Second, ISSA seeding is cost-saving from the perspective of seed materials. One small seed creates a much larger ESR than the original seed. Depending on the distance between mini-buffers, η ≳ 5, which clearly exceeds those attained from vertically connected seeding. Third, ISSA seeding with two-layer buffers protects YBCO crystals from seed-induced contamination. In contrast, without a buffer in between, Mg diffuses from the NdBCO/MgO seed to the crystal (Zhu *et al.*, 2020*a* ▸) in vertically connected seeding processes, degrading superconductivity in the vicinity of the seed. Nevertheless, the superior field trapping capability demonstrated above confirms that such local contamination has negligible influence here. Finally, for practical applications, ISSA seeding particularly suits bar-shaped YBCO crystals, which are traditionally fabricated using (100)/(100) arranged triple-seeds (Werfel *et al.*, 2012 ▸), with problems of contaminated grain boundaries. On the other hand, vertically connected seeds have potential applications for various shaped crystals in magnetic technology (*e.g.* the X pattern for square or hexagonal bulks).

Note that the calculated area ratios (η) in Fig. 11 ▸ are simply based on the individual growth habit of three categories, which do not reflect certain intricate growth behaviours. For instance, when a (110)-sided elongated seed was utilized (Nizhelskiy *et al.*, 2007 ▸), the growth habit changed during YBCO growth. Specifically, first the elimination of (110)-faces is not straightforward but arises by breaking up into steps with low-index (100) or (010) faces, yielding a serrated morphology in its early growth stage. Then, two neighbouring steps form a concave and an incomplete plane. Consequently, self-repair takes place from the concaves between steps and fills the gaps, combining small steps into big ones. By repeating the same behaviour, the rapid self-repairing evolution proceeds, in which a large number of small steps transform into a structure with a few large steps until all concaves are completed. As reported, under the same conditions, the induced YBCO crystals occupied 97% and 50% of the sample surface by utilizing (110)- and (100)-sided elongated seeds, respectively, indicating a faster growth generated by the (110)-sided seed.

## 4. Conclusions

Based on the natural function of self-repair, novel approaches have been developed to realize rapid *ab* plane coverage of YBCO crystals in TSMG for pursuing large size and high performance. Two constructions are designed to create non-equilibrium morphology with right-angled concave corners for triggering TSLG at two distinctive stages. One is *in situ* self-assembly seeding to attain biaxial in-plane alignment for promoting YBCO growth, while the other is vertically connected seeding to construct a precisely perpendicular arrangement for motivating YBCO nucleation. Through self-repair, crystals or seeds with shapes far from equilibrium induced rapid surface spreading to form initial growth morphology approaching equilibrium, which acted as a sizable effective seeding region to generate sizable equilibrium-shaped YBCO crystals. Consequently, benefiting from tailored structures, such as an enlarged *c*-GS, a fully grown surface, contamination-free grain boundaries and a suitable porosity, 25 mm- and 16 mm-diameter YBCO crystals exhibited superior field trapping capability in mono-peak profiles with record values of 0.8943 T and 0.7025 T, respectively. Finally, diverse seeding strategies with their growth habits and seeding effectiveness were reviewed, indicating that the novel complex seeding constructions in this work are promising for potential applications in TSMG of REBCO superconducting crystals.

For creating non-equilibrium morphology with self-reparability, newly designed seeding patterns are presented in the growth of YBa_2_Cu_3_O_7−δ_ crystals. Right-angled concave corners trigger rapid surface crystallization, which act as sizable effective seeding regions, generating tailored structures (an enlarged *c*-oriented growth sector, a fully grown surface, contamination-free grain boundaries and a suitable porosity) for superior field trapping capability.

## 5. Related literature

The following reference is cited in the supporting information: Kim *et al.* (2000 ▸).

## Supplementary Material

Supporting figures and equations. DOI: 10.1107/S2052252523000076/yc5041sup1.pdf
