# Supplementary material for: Natural strategies for creating non-equilibrium morphology with self-repairing capability towards rapid growth of excellent YBa2Cu3O7−δ crystals
Source: IUCrJ. 2023 Jan 25;10(Pt 2):177–88. doi: 10.1107/S2052252523000076 (PMC9980388; doi:10.1107/S2052252523000076)
Supplement: Supplementary file 1 [file m-10-00177-sup1.pdf]

IUCrJ

Volume 10 (2023)

Supporting information for article:

Natural strategies for creating non-equilibrium morphology with self-repairing capability towards rapid growth of excellent $YBa_2Cu_3O_{7-\delta}$ crystals

Yanhan Zhu, Yi Yang, Xiafan Gu, Qiang Gao, Pavel Diko and Xin Yao

1. Supplementary photographs of two crystals grown by precise and imprecise (110)/(110)-aligned seeding

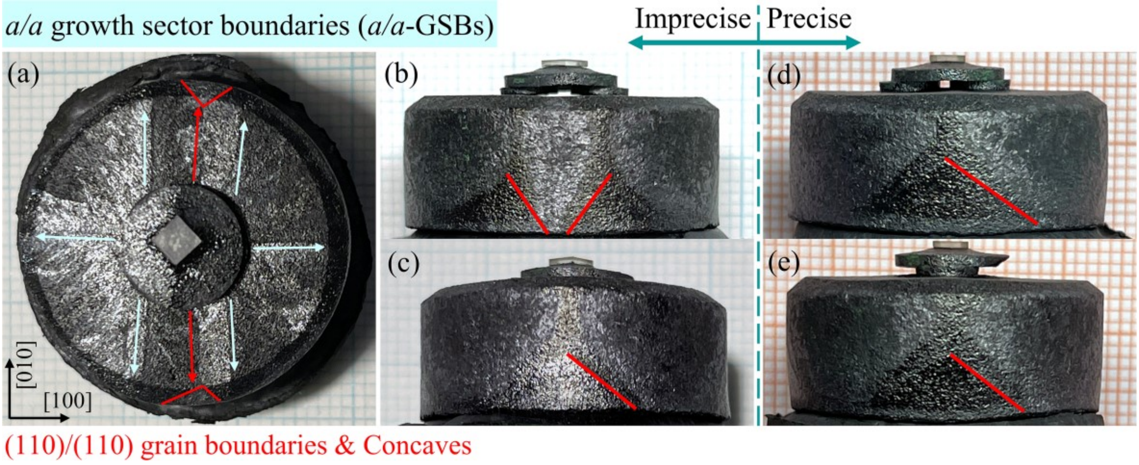

Figure S1. (a) Top view of a concave-remained YBCO crystal resulted from imprecise (110)/(110) aligned seeding, which possesses more *a*/*a*-GSBs and (110)/(110) grain boundaries. Side views of precise and imprecise (110)/(110) seeded crystals, (b,d) perpendicular and (c,e) parallel to the line connecting the centres of mini-buffers.

In contrast to the fascinating characteristics in Figure 4 (a-d) related to precise alignment, three totally different features appeared in Figure S1 (a) for the imprecise case, four (rather than two in Figure 4 (a)) facet lines (i.e., *a*/*a*-GSBs) parallel to the (110)/(110) grain boundary, invisible diamond-shaped region and discernible concaves at the sample edge. Characteristically, triangle regions are visible on all side views, which are known to be the manifestation of *c*-GS in a sample. A single triangle region appears in Figure S1 (d), verifying that two separate grains have combined into an integrated crystal in precisely aligned seeding. Notably, the height of triangle region is slightly higher in Figure S1 (d) than that in Figure S1 (e), signifying a faster crystallization of *a*-*b* plane in the corresponding direction and an enlarged *c*-GS. However, in imprecisely aligned seeding, although Figure S1 (c) has the same feature of a single triangle region like Figure S1 (e), Figure S1 (b) evidently exhibits two triangle regions instead of one in Figure S1 (d), proving the existence of two individual crystals. They induced a normal growth for two involved crystals, which neither yielded self-repairing nor brought about a completely combined crystal afterwards. Similar

observations were often reported from the prior art TSMG methods (Li *et al.*, 2010; Kim *et al.*, 2001; Kim *et al.*, 2000; Cheng *et al.*, 2013; Werfel *et al.*, 2012; Shi *et al.*, 2013).

2. Calculation of area ratios (η) of effective seeding region to the initial seed

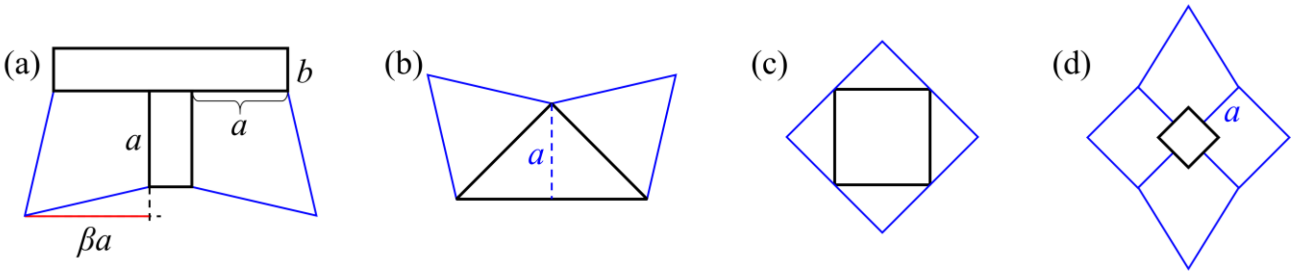

Figure S2. Schematic illustration of initial seeds (black lines) and their effective seeding regions (blue outlines).

In order to compare the seeding effectiveness of diverse seeding strategies, we introduce a parameter η that represents the area ratio of ESR to the initial seed. Figure S2 exhibits four representatives of all approaches. Before calculation, three ideal assumptions should be made. First, every corner has two edges of a in length. Second, the widths of strip-seeds in vertically-connected seeding are equally b. Third, the final edge length, as illustrated in Figure S2 (a), is βa with a fixed length ratio of β. Thus, every corner produces a same-sized kite-shaped region.

For vertically-connected patterns, the area of ESR (S_{ESR}) could be generally calculated by summing the area of seed (S_{seed}) and all kite-shaped regions (S_{kite}):

$$S_{ESR} = S_{seed} + N_{corner} \cdot S_{kite} \tag{S1}$$

where N_{corner} is the number of corners in a given type. Then, the area ratio of each ESR to its initial seed size could be easily obtained by

$$\eta = S_{ESR}/S_{seed}. \tag{S2}$$

Taking T-type as an example, the seed area is determined as

$$S_{seed}^{T} = (2a + b)b + ab = 3ab + b^2 \tag{S3}$$

while the area of corresponding ESR in Figure S2 (a) could be calculated as

$$S_{ESR}^{T} = 3ab + b^2 + 2\left(\frac{1}{2} \cdot a \cdot a\beta + \frac{1}{2} \cdot a \cdot a\beta\right) = 3ab + b^2 + 2\beta a^2 \tag{S4}$$

So,

$$\eta_T = \frac{3ab+b^2+2\beta a^2}{3ab+b^2} \quad \text{(S5)}$$

Similarly, the η results of other four types of vertically-connected seeding patterns can be derived as

$$\eta_Z = \frac{3ab+b^2+2\beta a^2}{3ab+b^2} \quad \text{(S6)}$$

$$\eta_L = \frac{2ab+b^2+\beta a^2}{2ab+b^2} \quad \text{(S7)}$$

$$\eta_X = \frac{4ab+b^2+4\beta a^2}{4ab+b^2} \quad \text{(S8)}$$

$$\eta_W = \frac{4ab+b^2+3\beta a^2}{4ab+b^2} \quad \text{(S9)}$$

For (110)-sided seeds, there are triangular, circular and square shapes. Based on the previous calculation, by drawing the blue dashed line in Figure S2 (b), the area of ESR of a triangular seed can be identified with that of two kite-liked regions from hypothetical corners. Accordingly, the area of ESR of a circular seed can be identified with that of four kite-liked regions from hypothetical corners. Their area ratios are calculated as

$$\eta_{triangular} = \frac{S_{ESR}}{S_{seed}} = \frac{2\beta a^2}{a^2} = 2\beta \quad \text{(S10)}$$

$$\eta_{circular} = \frac{S_{ESR}}{S_{seed}} = \frac{4\beta a^2}{\pi a^2} = \frac{4\beta}{\pi} \quad \text{(S11)}$$

Specially, for the square-shaped seed with all sides exposing (110) faces in Figure S2 (c), experimental result (Qian *et al.*, 2018) shows that its overgrowth manner is inhibited since all edges possess exactly equal lengths. Consequently, its initial growth morphology is equal to its equilibrium shape and

$$\eta_{square} = \frac{S_{ESR}}{S_{seed}} = 2 \quad \text{(S12)}$$

For ISSA seeding, the diamond region in Figure S2 (d) can also be regarded as two kite-liked areas with two square grains. The corner edge-length is $\sqrt{2}/2$ of the mini-buffer distance, i.e., $a = \sqrt{2}D/2$. The seed area (S_{seed}) is 2 × 2 mm^2 as mentioned in Experimental methods. Hence,

$$\eta_{ISSA} = \frac{S_{ESR}}{S_{seed}} = \frac{2a^2+2\beta a^2}{2\times 2} = \frac{(\beta+1)D^2}{4} \quad \text{(S13)}$$

Note that, in this strategy, η depends on the mini-buffer distance D (D = 3 mm for the η in Figure 11).

To give a direct numerical comparison, we put a = 4.5 mm, b = 2 mm and β = 1.3 into Equation (S5-S13) and derive all the area ratios depicted in Figure 11.

3. References

Li T. Y., Wang C. L., Sun L. J., Yan S. B., Cheng L., Yao X., Xiong J., Tao B. W., Feng J. Q., Xu X. Y., Li C. S. & Cardwell D. A. (2010). *J. Appl. Phys.* **108**, 023914.

Kim H.-J., Kim C.-J., Joo J.-H., Fuchs G. & Hong G.-W. (2001). *Physica C*, **357-360**, 899-902.

Kim C.-J., Kim H.-J., Jee Y. A., Hong G.-W., Joo J.-H., Han S.-C., Han Y.-H., Sung T.-H., Kim S.-J. (2000). *Physica C*, **338**, 205-212.

Cheng L., Guo L. S., Wu Y. S., Yao X. & Cardwell D. A. (2013). *J. Cryst. Growth*, **366**, 1-7.

Werfel F. N., Floegel-Delor U., Rothfeld R., Riedel T., Goebel B., Wippich D. & Schirrmeister P. (2012). *Supercond. Sci. Technol.* **25**, 014007.

Shi Y.-H., Durrell J. H., Dennis A. R., Cardwell D. A. (2013). *Supercond. Sci. Technol.* **26**, 015012.

Qian J., Ma L.-T., Du G.-H., Xiang H., Liu Y., Wan Y., Huang S.-M., Yao X., Xiong J. & Tao B.-W. (2018). *Scr. Mater.* **150**, 31-35.
